# Regularized Maximum Correntropy Criterion Kalman Filter for Uncalibrated Visual Servoing in the Presence of Non-Gaussian Feature Tracking Noise

**DOI:** 10.3390/s23208518

**Published:** 2023-10-17

**Authors:** Glauber Rodrigues Leite, Ícaro Bezerra Queiroz de Araújo, Allan de Medeiros Martins

**Affiliations:** 1 Electrical Engineering Department, Center of Technology, Federal University of Rio Grande do Norte—UFRN, Natal 59072-970, Brazil; allan@dee.ufrn.br; 2 Computing Institute, A. C. Simões Campus, Federal University of Alagoas—UFAL, Maceió 57072-970, Brazil; icaro@ic.ufal.br

**Keywords:** uncalibrated visual servoing, robot vision control, maximum correntropy criterion, non-gaussian kalman filtering

## Abstract

Some advantages of using cameras as sensor devices on feedback systems are the flexibility of the data it represents, the possibility to extract real-time information, and the fact that it does not require contact to operate. However, in unstructured scenarios, Image-Based Visual Servoing (IBVS) robot tasks are challenging. Camera calibration and robot kinematics can approximate a jacobian that maps the image features space to the robot actuation space, but they can become error-prone or require online changes. Uncalibrated visual servoing (UVS) aims at executing visual servoing tasks without previous camera calibration or through camera model uncertainties. One way to accomplish that is through jacobian identification using environment information in an estimator, such as the Kalman filter. The Kalman filter is optimal with Gaussian noise, but unstructured environments may present target occlusion, reflection, and other characteristics that confuse feature extraction algorithms, generating outliers. This work proposes RMCKF, a correntropy-induced estimator based on the Kalman Filter and the Maximum Correntropy Criterion that can handle non-Gaussian feature extraction noise. Unlike other approaches, we designed RMCKF for particularities in UVS, to deal with independent features, the IBVS control action, and simulated annealing. We designed Monte Carlo experiments to test RMCKF with non-Gaussian Kalman Filter-based techniques. The results showed that the proposed technique could outperform its relatives, especially in impulsive noise scenarios and various starting configurations.

## 1. Introduction

Sensing the surrounding environment is essential for a robot to perform its mission, from planning to control. It is common to see autonomous vehicles equipped with various, sometimes even redundant, sensors to perceive the environment, often relying on sensor fusion techniques [1]. Similarly, industrial robots require multiple sensors to work on strongly circumstance-dependent tasks, such as object grasping, obstacle avoidance, and human–robot collaboration [2]. These efforts show how crucial and difficult it is for a robot to understand the world, especially in unstructured environments, which have not been modified specifically to facilitate the execution of a task [3].

Cameras are devices containing a sensor that captures visual data in the form of images or video, which can be analyzed and processed to extract useful information about the environment. In the market, cameras are available at different costs and designs presenting sensors, such as RGB, RGB-D, and thermal sensors [4,5]. Some of the advantages of using cameras as sensor devices on feedback systems are the flexibility of the data it represents, the possibility to extract real-time information, and it does not require contact to operate.

Visual servoing is a control strategy that uses visual feedback from cameras to control the motion of a robot or a system. It has been extensively studied and developed over the past few decades and applied in various applications in robotics and automation, such as industrial robots [6], underwater vehicle manipulation [7,8], multiagent tasks [9], and medical imaging [10]. Visual servoing aims to use the visual information captured by the cameras to adjust a robot’s motion to achieve a desired task, such as positioning an object, following a path, or tracking a moving target. That visual information comes in the form of image features, which can be colored points, circle radius, bounding boxes, contours, or any image element of interest.

The visual servoing formulation relies on the mapping of image features and the camera pose, best described in terms of derivatives and an image jacobian matrix, also known as the interaction matrix, to relate them. However, this matrix requires knowledge of the intrinsic and extrinsic parameters of the camera, which are not easily obtained. Even when a camera calibration is made a priori, there are limitations to that approach, especially in a single-camera scenario and unstructured environments. Small physical disturbances to the device, non-linear lens distortion, and a tracking object moving out of the field of view are among the examples of situations requiring camera recalibration [11,12,13].

Uncalibrated visual servoing (UVS) aims to estimate the image jacobian matrix from offline preset moves or through online interactions. It may start with an initial guess from a previous calibration process or skip that phase entirely with random initial values. This approach permits a vision robotic system adaptable to the camera and environment changes. Despite the advances in computer vision, a recent review of vision-based robotic applications stated that images have faults due to problems like picture noise [14]. If that noise is not properly treated, it can lead to difficulties in the image Jacobian estimation convergence.

The examples shown previously, which would require camera recalibration, can be treated as noise in the image features tracking task. Moreover, other sources of noise can be changing light conditions and motion blur. As UVS adaptively estimates the interaction of features and camera pose, most algorithms can handle the uncertainty if the noise is small. There are techniques concerning UVS estimation in the presence of Gaussian noise that may work well under smooth and well-behaved disturbances, like camera vibration and motion blur. Nonetheless, impulsive and non-Gaussian noise occurs when, for example, a tracking object suddenly moves out of the field of view, or the light in the image is interfered momentarily by other light sources, producing reflections.

Generally, algorithms expecting non-Gaussian noise work well with Gaussian noise, but not the other way around [15]. Some of the techniques to estimate non-Gaussian noise are particle filter, M-estimator, and Maximum Correntropy Criterion (MCC). A convenience of MCC is that it takes advantage of the probabilistic properties of the non-Gaussian random variables, as it uses the correntropy similarity metric, associating that to an optimization problem. Approaches presented in [16,17,18] aim to implement the MCC in the Kalman Filter.

Using the traditional MCC with the Kalman Filter formulation, in ref. [19], online interaction matrix estimation was implemented, relating feature motion to a robot-attached camera motion. While two non-Gaussian noise scenarios were studied, it is possible to develop a more extensive study in the characterization of that noise in the UVS application. Furthermore, while that study determines camera-to-joint motion using robot geometry, a general estimation to map feature motion directly to joint motion is possible. Although most IBVS tasks present more than one image feature, the reviewed techniques skip the correction step when a single feature presents an outlier measure, keeping the jacobian estimation from the previous iteration, which may not correspond to the system’s current state.

This work proposes a different approach for Maximum Correntropy Criterion Kalman Filtering, designed specifically for applying single-camera uncalibrated image-based visual servoing, where the IBVS control law is affected by a correntropy-induced error vector. It uses a regularization term to ensure that matrices used in the correction step remain non-singular, thus maintaining the estimation update even when some features are detected as outliers by using the values from other features. Also, we propose a simulated annealing to adjust the MCC kernel bandwidth, which regulates the tolerance of the estimation update strategy to large estimation errors that could be caused by measure outliers. Our approach estimates the whole feature to joint space jacobian matrix, handling robot geometry modeling uncertainties through online data. The technique was extensively tested using Monte Carlo experiments, varying robot starting configurations, and applying additive impulsive noise distributions.

This work is organized as follows: Section 2 presents fundamentals on Uncalibrated Visual Servoing (UVS) and introduces Kalman Filter, how it can be seen as an optimal estimator for state observing, and how it can be applied to UVS. Section 3 explores the problem and efforts of dealing with non-Gaussian noise on UVS, and how the Maximum Correntropy Criterion is used to address that in the form of two main techniques, MCKF and IMCC-KF. Section 4 brings the main contribution of this work, describing the problems of the previously presented techniques in the studied context and proposing algorithms designed specifically for it. Section 5 shows the methodology of the experiments designed to test the proposed technique’s performance, including the system architecture, simulation scenarios, and Monte Carlo experiments. Section 6 discusses the results in three comparison scenarios. Section 7 concludes this work, presenting limitations and future work.

**Notation** **1.** *Throughout this paper, we introduce some notation rules for clarity. Bold lower case letters denote vectors, bold upper case letters are matrices. Symbol ∧ above a matrix, vector, or scalar represents an estimate of the counterpart. In addition, we define notation 0 to denote a zero matrix.*

## 2. Uncalibrated Visual Servoing

**Figure 1:**
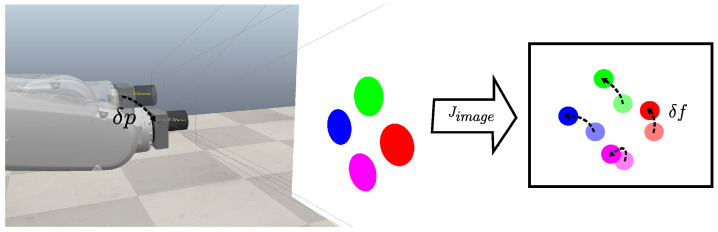
Relation between camera and image motion described by the image jacobian. In an eye-in-hand IBVS configuration, the robot’s joint displacement moves the camera and, consequently, the position of the features in the image frame.

In image-based visual servoing (IBVS) control architecture, where robot motion action is computed directly by image information, image jacobian $J_{image}$ describes the relationship between feature displacement δf and camera velocity δp, as described in Figure 1. Thus, control action v(k+1) is designed to compute the required camera velocity based on the error between the desired feature values $f_{d}\left(k\right)$ and the measured $f_{m}\left(k\right)$, and a gain λ∈R, as shown in Equations (1) and (2).

(1) $$\begin{array}{cc} \mathrm{error}(k) & =f_{m}\left(k\right)-f_{d}\left(k\right), \end{array}$$

(2) $$\begin{array}{cc} v(k+1) & =-\lambda {J_{\mathrm{image}}}^{†}\left(k\right)\mathrm{error}\left(k\right), \end{array}$$

where ${J_{\mathrm{image}}}^{†}\left(k\right)$ is the pseudoinverse of $J_{\mathrm{image}}\left(k\right)$. As described in the basics of image formation [12], the image jacobian varies given the position of the tracking objects in the real world and the camera. When the camera is attached to the robot end-effector, in an eye-in-hand configuration, the motion of robot joints is important for the IBVS. Thus, a general IBVS control law u(k+1) should consider the robot kinematics jacobian $J_{\mathrm{kine}}$ that relates the end-effector motion to joint velocities and the image jacobian matrix, as shown in Equations (3) and (4).

(3) $$\begin{array}{cc} J_{\mathrm{full}}\left(k\right) & =J_{\mathrm{image}}\left(k\right)J_{\mathrm{kine}}\left(k\right), \end{array}$$

(4) $$\begin{array}{cc} u(k+1) & =-\lambda {J_{\mathrm{full}}}^{†}\left(k\right)\mathrm{error}\left(k\right). \end{array}$$

Estimating the image jacobian is the main concern in many uncalibrated visual servoing problems, but there are advantages in using the estimation for the full jacobian. The first advantage is that, as estimation relies on data through iteration, it can handle uncertainties in robot modeling. Secondly, the IBVS algorithm can skip the computation of teh $J_{\mathrm{kine}}$ matrix and the matrix multiplication of Equation (3). On the other hand, estimating the full jacobian can be harder than estimating the image jacobian, so it is preferable to use an analytical initial guess at the start of the task, through camera calibration and geometric robot modeling, such as Denavit–Hartenberg parameters.

Among the practical considerations related to uncalibrated visual servoing tasks [20], the error produced by the visual feature tracker should be handled with care. To maintain high precision, visual tracking algorithms are often computationally expensive, which may provide delays in feedback for the entire control system, affecting the whole system’s performance. In contrast, the traditional image-based visual servoing approach is known to be quite robust to image jacobian uncertainty [12], but dealing with feedback noise effectively requires special considerations in the algorithm formulation. Because of that, Kalman filtering estimation is a recurring approach in UVS studies.

### Kalman Filter for UVS

The Kalman filter is a mathematical algorithm introduced as an attempt at a recursive solution to the discrete-data linear filtering problem [21], and it has become one of the most popular and powerful algorithms in the field of control theory. It takes measurements of a system’s state and combines them with predictions of the system’s state to produce an estimate that is as accurate as possible. Two main stages compose the algorithm: the prediction stage, which estimates using a mathematical model, and the correction stage, which improves the estimation using new measurements.

At its core, the Kalman filter is an optimal estimator that can be seen as a state observer that handles uncertainty [22]. We consider a causal linear discrete system in the state-space formulation, given the *k*th iteration, with state vector x, input vector u, state transition model F, input model G, and observation model H:

(5) $$\begin{array}{cc} x(k+1) & =F(k)x(k)+G(k)u(k), \end{array}$$

(6) $$\begin{array}{cc} y(k) & =H(k)x(k). \end{array}$$

That dynamical system presents no uncertainty in the process or measure equations. Thus, a simple observer can be designed to track the system’s evolution, using prediction $\hat{x}\left(k+1|k\right)$ given the actual state and input, and a set of states Ω consistent with measurement y(k+1). This implies an estimated state $\hat{x}\left(k+1|k+1\right)$ that corrects $\hat{x}\left(k+1|k\right)$ to the set Ω using Δx. The choice of Δx is related to an optimal gain K times an innovation error ν defined as the difference between the measured output y(k+1) and the predicted output $H\left(k+1\right)\hat{x}\left(k+1|k\right)$. In that case, that gain is calculated using the fact that for Δx to be the shortest path to Ω, it should be orthogonal.

This fact does not sustain, as the modeled process equation for x(k+1) and measures y(k) can be uncertain. That is the case in which the Kalman filter is generally presented to the full extent. Considering that uncertainty values present zero-mean Gaussian noise, the uncertainty variable for the process ν(k), and in the measurement w(k), present covariances V(k) and W(k), respectively. Thus, the Kalman filter expects a system described by the following expressions:

(7) $$\begin{array}{cc} x(k+1) & =F(k)x(k)+G(k)u(k)+\nu (k), \end{array}$$

(8) $$\begin{array}{cc} y(k) & =H(k)x(k)+w(k). \end{array}$$

Also, vectors ν(k) and w(k) should be independent random variables. Moreover, using E{·} as the expected value operator and cov{·,·} as the covariance function, the probabilistic conditions of the Kalman filter are summarized below:

(9) $$\begin{array}{cc} E\{\nu (k)\} & =0, \end{array}$$

(10) $$\begin{array}{cc} E\{w(k)\} & =0, \end{array}$$

(11) $$\begin{array}{cc} \mathrm{cov}\{\nu (k),\nu (k)\} & =E\{\nu \left(k\right)\nu {\left(k\right)}^{⊤}\}=V\left(k\right), \end{array}$$

(12) $$\begin{array}{cc} \mathrm{cov}\{w(k),w(k)\} & =E\{w\left(k\right)w{\left(k\right)}^{⊤}\}=W\left(k\right), \end{array}$$

(13) $$\begin{array}{cc} \mathrm{cov}\{\nu (k),w(k)\} & =E\{\nu \left(k\right)w{\left(k\right)}^{⊤}\}=0. \end{array}$$

The predicted state $\hat{x}\left(k+1|k\right)$ is computed using the model dynamics as if there is no noise in the system. The filter predicts the error covariance P(k+1|k) using the definition of the covariance matrix expectation. Equations (14) and (15) present the prediction steps in the Kalman filter. The filter receives measure y(k+1), modeled with white noise w(k+1) that has covariance W(k+1). Then, it computes an optimal gain K using Equation (16). That gain is important to update the estimative on $\hat{x}\left(k+1|k+1\right)$ and P(k+1|k+1), as shown in Equations (17) and (18), respectively. Particularly in the state update equation, gain K weights the components of the innovation error, directing to where the state should correct its prediction.

(14) $$\hat{x}\left(k+1|k\right)=F\left(k\right)\hat{x}\left(k|k\right)+G\left(k\right)u\left(k\right),$$

(15) $$P\left(k+1|k\right)=F\left(k\right)P\left(k|k\right)F{\left(k\right)}^{⊤}+V\left(k\right),$$

(16) $$K=P\left(k+1|k\right)H{\left(k+1\right)}^{⊤}{\left(H\left(k+1\right)P\left(k+1|k\right)H{\left(k+1\right)}^{⊤}+W\left(k+1\right)\right)}^{-1},$$

(17) $$\hat{x}\left(k+1|k+1\right)=\hat{x}\left(k+1|k\right)+K\left(y\left(k+1\right)-H\left(k+1\right)\hat{x}\left(k+1|k\right)\right),$$

(18) $$P\left(k+1|k+1\right)=\left(I-KH\left(k+1\right)\right)P\left(k+1|k\right){\left(I-KH\left(k+1\right)\right)}^{⊤}+KW\left(k\right)K.^{⊤}$$

The Kalman filter provides an approach that can efficiently handle Gaussian noise in the online estimation of the image jacobian. The works using that approach treat the estimation process as an evolving linear system with associated probabilistic uncertainty. A state vector is presented by concatenating the interaction matrix’s estimated row elements, and the jacobian is estimated through measures of how the features change over time compared to the end-effector motion, so that $\hat{x}\left(k|k\right)$ is a flattened version of $J_{image}\left(k\right)$ or $J_{full}\left(k\right)$. Thus, the uncertainty is present in how the jacobian estimation evolves and in the measurement of the features, being treated as random variables with Gaussian distribution.

The main assumptions of the traditional Kalman filter are that the system has linear dynamics and the uncertainties in its process and measure follow Gaussian distributions. Although the problem of uncalibrated visual servoing is known to be nonlinear, most studies make approximations to assure linearity, which works best with slow dynamics. Robot motion may not be slow for human perception, but mechanical movement can be considered slow for the algorithm loop if the computation power is high enough. Furthermore, as initially proposed, the Kalman filter is robust to modeling errors at a certain level because it handles process uncertainty. On the other hand, non-Gaussian noise in the measuring step is very likely to appear, especially in unstructured and uncontrolled environments, where approximations may interfere with task performance.

## 3. Dealing with Non-Gaussian Noise

A simple mission for humans, such as picking fruit from an orchard, could present a challenging problem to computer vision algorithms. During the manipulation task, leaves can suddenly occlude the fruit, removing it from the image frame. If the task is being performed in the field, a cloud in the sky could pass before the sun, degenerating the scene’s illumination. Different fruits can present different textures that may present reflection to light, which can affect processing image steps. Finally, the sudden presence of some other object with similar image properties can confuse the feature tracking. All these are examples of situations where feature tracking can present non-Gaussian behavior, with outliers from a normal distribution.

While outliers can be disregarded using high-pass filters or more complex computer vision treatments, there are situations where the presence of these outliers is significant. For example, these outliers can sometimes be well described by distribution models, like a Gaussian mixture, preserving the statistical properties of the variable. However, the Kalman filter is not prepared to properly handle variables presenting that characteristic. Thus, the Kalman filter would consider the variable to be a Gaussian distribution, where the effect of the outlier is to offer a wrong average as the first-order expected value, and the correction step, which relies on second-order covariance matrices to compute the optimal gain, would not guarantee its optimality.

In paper [23], UVS with particle filter jacobian estimation was proposed where fuzzy membership functions adaptively change the number of particles. However, that work simulated a holonomic mobile planar robot, which presents no constraints in movement. In study [24], an iteratively reweighted least-square algorithm to UVS was proposed, implementing an M-estimator that optimizes a residual function. On the other hand, this method requires a memory of pairs involving the feature vector and the respective motor positions vector to aid the estimation, using the K-nearest neighbor pairs to estimate the jacobian for the current state the system is at.

Lately, in [19], the Maximum Correntropy Criterion was applied to the correction step in the Kalman filter correction; it uses correntropy, a similarity metric for random variables based on their whole probabilistic distribution. There is no need for memory since the algorithm is recursive. As that metric is insensitive to large errors, it is suitable for estimation in the presence of outliers, for example. However, the estimation algorithm used in that work, known as MCKF, as it was originally proposed, avoids the estimation of the whole jacobian if one of the tracking features presents an outlier, leading to potential problems in the IBVS control law. Also, an extension of that study could be applying the estimation for the whole jacobian matrix shown in Equation (3), instead of only the image jacobian. This work addresses these disadvantages with a new formulation of IBVS and jacobian estimation using the Maximum Correntropy Criterion and the Kalman Filter.

### 3.1. Maximum Correntropy Criterion

The Maximum Correntropy Criterion (MCC) can take advantage of the statistical properties of non-Gaussian random variables. Unlike traditional estimation criteria, MCC aims to maximize the correntropy between the observed data and the model prediction. The correntropy function measures the similarity between two signals by considering the nonlinear dependencies and higher-order statistical moments. Given two random variables X and Y, with joint distribution $F_{XY}\left(x,y\right)$, correntropy is defined by Equation (19).

(19) $$V\left(X,Y\right)=E\left\{\kappa \left(X,Y\right)\right\}=\int \kappa \left(x,y\right)dF_{XY}\left(x,y\right).$$

Kernel function κ(·,·) determines the shape of the correntropy measure. While several kernel options exist, which are shift-invariant symmetric positive-definite functions that follow Mercer’s theorem, the Gaussian kernel is a popular choice in correntropy-based problems because of its favorable mathematical properties. Considering *e* as the difference between the random variables, and a kernel bandwidth σ>0, the Gaussian kernel is given by Equation (20).

(20) $$\kappa \left(x,y\right)=G_{\sigma }\left(e\right)=\exp\left(-\frac{e^{2}}{2\sigma^{2}}\right).$$

The Gaussian kernel has a bell-shaped curve that assigns higher weights to points closer to the curve’s center and lower weights to points farther away. This makes it suitable for measuring the similarity between two signals, as it offers more weight to similar points.

A model estimation problem can implement the MCC to maximize the correntropy between the observed data and the model prediction. Given a sequence of N error data and the problem of learning a parameter vector W of a model from a set of feasible parameters Ω, an MCC-based estimation is the solution to the optimization problem shown in Equation (21).

(21) $$\hat{W}=\underset{W\in \Omega }{\arg\max}\frac{1}{N}\sum_{i=1}^{N}G_{\sigma }\left(e\left(i\right)\right).$$

Therefore, MCC effectively increases the value of the error probability density function at zero, which is the natural thing to do in regression or adaptive filtering, where the goal is to increase the number of small deviations between X and Y [25]. Parameter estimation implementations using the Maximum Correntropy Criterion can solve the optimization problem using iterative methods, such as fixed-point iteration.

### 3.2. Maximum Correntropy Kalman Filter

A derivation of the Kalman Filter to use MCC instead of the Minimum Mean Square Error (MMSE) criterion was proposed to perform better in non-Gaussian noise scenarios; that technique is called a Maximum Correntropy Kalman Filter (MCKF) [16]. It uses the same prediction steps as the traditional Kalman Filter, using model propagation equations to estimate the state and the covariance matrix. On the other hand, the MCKF update step uses the correntropy of the innovation error in a Gaussian kernel as an optimization criterion to weigh the correction in the posterior estimation. This approach considers a Kalman linear model, as shown in Equation (22).

(22) ϕ(k)=Ψ(k)x(k)+e(k).

This equation is obtained from the system model in Equations (7) and (8) through transformations and matrix decompositions using the following expressions:

(23)x^(k|k−1),y(k)=IH(k)x(k)+η(k),(24)η(k)=−(x(k)−x^(k|k−1))w(k),(25)E{η(k)η(k)⊤}=P(k|k−1)00W(k)(26)=Bp(k|k−1)Bp(k|k−1)⊤00Bw(k)Bw(k)⊤(27)=B(k)B(k)⊤,(28)ϕ(k)=B−1(k)x^(k|k−1)y(k),(29)Ψ(k)==B−1(k)IH(k),

where the state vector x has *n* values, the measurement vector y has m values, I is a n×n identity matrix and B is obtained using Cholesky decomposition.

From this formulation, an MCC optimization problem can be defined using Equation (30), where a Gaussian kernel with bandwidth σ evaluates the similarity between the variables composing the innovation error from Equation (22). That cost function gives the optimal estimate to x(k), serving as an update step in this derivation of the Kalman filter. That equation uses $e_{i}\left(k\right)$ as the ith row of e(k), $\phi_{i}\left(k\right)$ as the ith row of ϕ(k), and $\psi_{i}\left(k\right)$ as the ith row of Ψ(k).

(30) $$\hat{x}\left(k\right)=\underset{x(k)}{\arg\max}\sum_{i=1}^{n+m}G_{\sigma }\left(e_{i}\left(k\right)\right)=\underset{x(k)}{\arg\max}\sum_{i=1}^{n+m}G_{\sigma }\left(\phi_{i}\left(k\right)-\psi_{i}\left(k\right)x\left(k|k-1\right)\right).$$

An algorithm using fixed-point iteration can solve Equation (30). We let t=1 and $\hat{x}{\left(k|k\right)}_{0}=\hat{x}\left(k|k-1\right)$, where $\hat{x}{\left(k|k\right)}_{t}$ describes the estimated state at fixed-point iteration *t*, computed as follows:

(31) $$e_{i}\left(k\right)=\phi_{i}\left(k\right)-\psi_{i}\left(k\right)\hat{x}{\left(k|k\right)}_{t-1},$$

(32) $$C_{x}\left(k\right)=\mathrm{diag}(G_{\sigma }\left(e_{1}\left(k\right)\right),\cdots ,G_{\sigma }\left(e_{n}\left(k\right)\right),$$

(33) $$C_{y}\left(k\right)=\mathrm{diag}(G_{\sigma }\left(e_{n+1}\left(k\right)\right),\cdots ,G_{\sigma }\left(e_{n+m}\left(k\right)\right),$$

(34) $$P\left(k|k-1\right)=B_{p}\left(k|k-1\right){C_{x}}^{-1}\left(k\right)B_{p}\left(k|k-1\right){,}^{⊤}$$

(35) $$W\left(k\right)=B_{w}\left(k\right){C_{y}}^{-1}\left(k\right)B_{w}{\left(k\right)}^{⊤},$$

(36) $$K=P\left(k|k-1\right)H{\left(k\right)}^{⊤}{\left(H\left(k\right)P\left(k|k-1\right)H{\left(k\right)}^{⊤}+W\left(k\right)\right)}^{-1},$$

(37) $$\hat{x}{\left(k|k\right)}_{t}=\hat{x}\left(k|k-1\right)+K\left(y\left(k\right)-H\left(k\right)\hat{x}\left(k|k-1\right)\right).$$

The fixed-point iteration algorithm requires a stop condition, so that if a new iteration does not bring relative contribution to the estimation, the algorithm stops. That condition is shown in Equation (38), where the small positive ε holds the threshold to either stop the algorithm or demand a new iteration, with t←t+1. If a new iteration is required, the algorithm calculates Equations (31)–(37), starting with the state vector estimation from the previous iteration.

(38) $$\frac{\left|\right|\hat{x}{\left(k|k\right)}_{t}-\hat{x}{\left(k|k\right)}_{t-1}\left|\right|}{\left|\right|\hat{x}{\left(k|k\right)}_{t-1}\left|\right|}\leq \varepsilon .$$

The covariance matrix update is computed afterward, using the optimal gain K. While calculating K differs from the traditional Kalman filter, the covariance matrix correction uses the same Equation (18). If an error component $e_{i}\left(k\right)$ presents a very large value, it indicates an outlier, so $C_{x}$ or $C_{y}$ can become singular. When that happens, the algorithm stops, and the correction step should be skipped, retaining the last estimation, so $\hat{x}\left(k|k\right)=\hat{x}\left(k|k-1\right)$.

### 3.3. Improved Maximum Correntropy Criterion Kalman Filter

Based on the one iteration fixed point approach suggested on [17], which implies simplifications in the correction equations, in ref. [18], an algorithm was proposed called the Improved Maximum Correntropy Kalman Filter (IMCC-KF). The goal was to improve estimation accuracy while retaining the computational complexity. It applied a correntropy-induced algorithm where the $W^{-1}$ norm of the estimation innovation error is evaluated through a kernel function, resulting in a scalar γ(k).

(39) $$e\left(k\right)=y\left(k\right)-H\left(k\right)\hat{x}\left(k|k-1\right),$$

(40) $$\gamma \left(k\right)=G_{\sigma }\left(\sqrt{e{\left(k\right)}^{⊤}W^{-1}e\left(k\right)}\right),$$

(41) $$W_{e}=\gamma \left(k\right)H\left(k\right)P\left(k|k-1\right)H{\left(k\right)}^{⊤}+W,$$

(42) $$K=\gamma \left(k\right)P\left(k|k-1\right)H{\left(k\right)}^{⊤}{W_{e}}^{-1},$$

(43) $$\hat{x}\left(k|k\right)=\hat{x}\left(k|k-1\right)+K,\left(y\left(k\right)-H\left(k\right)\hat{x}\left(k|k-1\right)\right).$$

When there is no big error between the estimated and the measured value, i.e., innovation error, γ(k) tends to 1 and the algorithm is reduced to a Kalman filter. Otherwise, if e(k) is sufficiently big, it could imply that the measured value is an outlier, leading γ(k) to 0, as well as the gain K, so there is no update. That approach follows the same analysis that if the kernel bandwidth σ→∞, it reduces to a traditional Kalman Filter, for all measured values.

## 4. Regularized Maximum Correntropy Criterion Kalman Filter for IBVS

Using the base formulation of MCKF and the single-iteration considerations of IMCC-KF, we developed a correntropy-induced technique for Kalman Filtering, specifically for the IBVS jacobian estimation and the control law. Therefore, the algorithm actuates in two phases of the visual servoing system. To better illustrate the case in which the proposed approach was designed, we consider an IBVS problem of tracking four circles, each circle presenting two features for the XY coordinates of its center. Due to light problems, occlusion, or detection failure, the vision system states that the circle is temporarily in a very distant spot. In that situation, the covered techniques should detect two features with large estimation errors, such as a potential outlier, and skip the correction step, preserving the last estimated value for the jacobian. This impacts the control law since it tries to minimize the error in the feature space using wrong values for the system’s current state. That way, the correctly measured features from the remaining circles are discarded.

Using the previously presented MCKF, the matrix $C_{y}\left(k\right)$ degenerates since it is a diagonal matrix and some of its diagonal values are close to zero. This makes $C_{y}\left(k\right)$ singular, resulting in the algorithm skipping the estimation update for all features in the iteration. To avoid terminology confusion, we define

(44) $$E\left(k\right)=\mathrm{diag}(G_{\sigma }\left(e\left(k\right)\right)),$$

which is positive semi-definite, and could be singular. However, we can apply regularization term $\lambda_{r}>0$ to enforce a condition to ensure matrix inversion. Since E(k)⪰0, we have

(45) $$(E\left(k\right)+\lambda_{r}^{2}I)≻0$$

that is always positive definite and, consequently, invertible. Therefore, if one of the eigenvalues of E(k) is zero, its respective inverting Equation (45) will be ${\left(\lambda_{r}^{2}\right)}^{-1}$. In the context of the present work, since another inversion will happen to compute the optimal gain K, it means that only parts of the jacobian estimation respective to that problematic feature will not be updated.

Algorithm 1 estimates the jacobian based on how feature measures change, compared to how the robot joints update. Symbol ⨂ is the kronecker matrix product operator. One of the main advantages of this approach, in the context of IBVS, is that while the other approaches skip the whole correction step if any feature presents a potential outlier measurement, the proposed algorithm tries to update the jacobian values based on the trusted measures only, depending on the kernel bandwidth σ, skipping only parts of the jacobian related to the problematic measures.

The regularization in Line 9 allows the algorithm continuation of computing a value for $\hat{R}$ and, consequently, K. Considering $\lambda_{r}$ small, the effect of the outlier measure in the estimation process is reduced to $\lambda_{r}^{2}$, which does not harm the remaining measures. If the tracking of the *i*th feature is consistent with its estimation, $e_{i}\left(k\right)\to 0$ and $G_{\sigma }\left(e_{i}\left(k\right)\right)\to 1$, then the correction step works almost like the Kalman Filter.

Algorithm 2 uses the resulting estimation from Algorithm 1 in an outer loop, calculating the robot joint velocities that lead the image features to their desired position. If only the RMCKF is used with the traditional IBVS control law, from Equation (4), a problematic feature tracking measure suppressed in the RMCKF still impacts the feature error computation and produces undesired robot motion. Thus, it is desirable that the jacobian or the error components relative to that outlier should have a close to zero effect in the control law, but not stop the whole robot motion for the remaining features. The proposed algorithm solves that by incorporating the correntropy-induced vector $G_{\sigma }\left(e\left(k\right)\right)$ into the error component, using ⨀ as the element-wise multiplication operator. If the jacobian components were altered instead, the last trusting estimation would be lost, potentially affecting the estimation algorithm convergence.
**Algorithm 1** Regularized Maximum Correntropy Criterion Kalman Filter**Ensure:**  κ(·)⪰0▹ Kernel follows Mercer Theorem**Require:**  $f_{m}\left(k\right)$▹ Reading of current features values**Require:**  q(k)▹ Reading of current motor joint positions  1: **procedure** RMCKF (σ)  2:     x(k|k−1)←x(k−1|k−1)  3:     P(k|k−1)←P(k−1|k−1)+V  4:    $Z\left(k\right)\leftarrow f_{m}\left(k\right)-f_{m}\left(k-1\right)$▹ Measurement may be contaminated with noise  5:     H(k)←I⨂(q(k)−q(k−1))  6:     $B_{R}\leftarrow \mathrm{cholesky}\left(R\right)$▹ Cholesky matrix decomposition  7:     $e\left(k\right)\leftarrow {B_{R}}^{-1}Z\left(k\right)-{B_{R}}^{-1}H\left(k\right)x\left(k|k-1\right)$  8:     $E\left(k\right)\leftarrow \mathrm{diag}(G_{\sigma }\left(e\left(k\right)\right))$  9:     $\hat{R}\leftarrow {B_{R}}^{⊤}{\left(E\left(k\right)+\lambda_{r}^{2}I\right)}^{-1}B_{R}$10:     $K\leftarrow P\left(k|k-1\right)H{\left(k\right)}^{⊤}{\left(H\left(k\right)P\left(k|k-1\right)H{\left(k\right)}^{⊤}+\hat{R}\right)}^{-1}$11:     x(k|k)←x(k|k−1)+K(Z(k)−H(k)x(k|k−1))12:     $P\left(k|k\right)\leftarrow {\left(I-KH\left(k\right)\right)P\left(k\right)|k-1)\left(I-KH\left(k\right)\right)}^{⊤}+KRK^{⊤}$13:     **return** x(k|k)▹ Returns the estimation, other variables persist for the next loop14:  **end procedure**
**Algorithm 2** IBVS with RMCKF**Require:**  λ▹ IBVS gain**Require:**  $t_{max}$▹ IBVS end loop time**Require:**  $T_{s}$▹ Discrete timestep**Require:**  $\sigma_{last}$▹ Kernel bandwidth  1: t←0  2: k←0  3: **while** 
$t<t_{max}$
 **do**  4:     **if** annealing = TRUE **then**  5:         $\sigma \leftarrow \sigma_{last}+100\left(1-\frac{kT_{s}}{t_{max}}\right)$  6:     **else**  7:         $\sigma \leftarrow \sigma_{last}$  8:     **end if**  9:     x(k|k)←RMCKF(σ)10:     J(k)←reshape(x(k|k))▹x(k|k) is a flat version of J(k)11:     $\mathrm{error}\left(k\right)\leftarrow f_{m}\left(k\right)-f_{d}\left(k\right)$12:     $u\left(k+1\right)\leftarrow -\lambda J{\left(k\right)}^{†}\left(G_{\sigma }\left(e\left(k\right)\right)⨀\mathrm{error}\left(k\right)\right)$▹ Correntropy-induced IBVS law13:     k←k+114:     $t\leftarrow t+T_{s}$15:  **end while**

As implemented with the other approaches tested in this work, a simulated annealing implementation for σ is shown in Lines 4 to 8. In the context of UVS, that is a useful resource, since a fixed small kernel bandwidth can result in an estimation convergence that is too slow. That annealing, which is exclusive in this work, aims to make the RMCKF work closer to a traditional Kalman Filter at the beginning of the task when the algorithm should expect more learning on the difference between the measures and the correspondent estimated motion. As the task evolves, the algorithm can use a small σ, so it still learns but becomes more intolerant to impulsive changes in the estimation. The Results section provides a comparison of using the simulated annealing and establishing a fixed kernel bandwidth.

In summary, the three main concerns that the proposed algorithm aims to solve are the following: (1) The correction step for the jacobian estimation should consider the features with reliable measurements and ignore the untrusted ones, not skip entirely if one single measure is a potential outlier; (2) While the IBVS control action should disregard motion for the unreliable measures, the robot should not stop entirely, since it can try to minimize the error components of the remaining measures; (3) At the beginning of the task, the estimation algorithm can be more permissive to bigger changes, but as time goes on, it should become more resistant to those as the estimation would likely be closer to convergence and those impulsive changes have a great chance to be measurement outliers.

## 5. Materials and Methods

**Figure 2:**
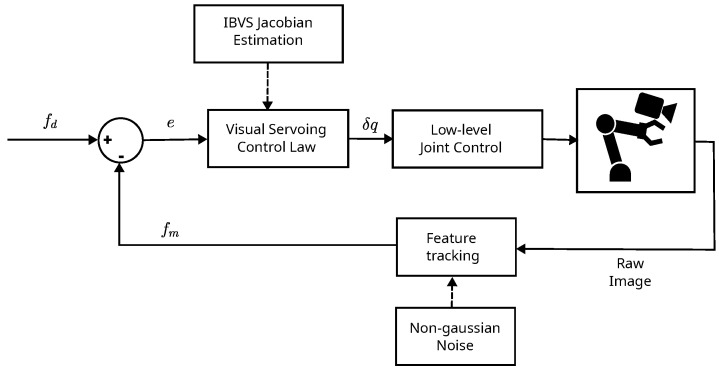
Overview of the proposed visual servoing system.

The visual servoing system which is being studied and proposed in this work is in the category of image-based visual servoing (IBVS), which uses the features of the image directly in the feedback. The desired image features values $f_{d}$ are compared to the measured features $f_{m}$, as shown in Figure 2, which depicts the system overview. That comparison generates the error signal error, applied in the visual servoing control law to compute the resulting robot joint motion u. The visual servoing control law requires the IBVS jacobian that is being estimated either from a fixed approximation using the application of image formation theory and robot kinematics modeling equations in Equation (3) or through the use of recursive techniques, i.e., traditional KF, MCKF, IMCCKF or the proposed RMCKF. As this study aims primarily at eye-in-hand visual servoing configuration, the camera pose is the same as the robot’s end-effector pose.

The robot is assumed to have an inner control loop to receive joint position and velocity commands. Thus, at the start of each loop, the system reads the current robot joint position q(k). Furthermore, a computer vision algorithm extracts and matches features to track how they change between image frames. To simulate the problems in real unstructured scenarios, the system adds non-Gaussian noise to feature tracking using an independent random number generator for each feature, following a proper impulsive probabilistic distribution known as the Lévy α-stable distribution.

### 5.1. Simulation

The UVS scenarios were simulated using the CoppeliaSim robotics simulator, which implements the Bullet physics engine. That tool allows us modelling of a robot, control of its joints, placement of cameras attached to or outside the robot structure, and seting of different parameters on physics, image acquisition, and environment. CoppeliaSim provides an Application Programming Interface (API) in various programming languages to implement custom control algorithms. This study used the Python programming language since its numerical and scientific libraries offer fast prototyping for the discussed techniques.

A Universal Robots UR10 was placed in the simulation using one of the predefined models in CoppeliaSim. This robot was chosen by a correlated study, which described a UVS system using MCKF [19]. Since this work estimates the conjoined jacobian that relates image features and joint movement directly, a kinematic description of the robot isprovided. The Denavit–Hartenberg parameters for the UR10 are presented in Table 1, using the standard formulation and link sizes obtained from the official technical specifications. Since all robot joints are revolute, the robot configuration is defined by vector $q=\{\theta_{1},\;\theta_{2},\;\theta_{3},\;\theta_{4},\;\theta_{5},\;\theta_{6}\}$; thus, the joints are configured in the Kinematic mode to receive joint position commands. The offset values of the $\theta_{i}$ column were defined to match the joint commands to the simulated robot’s joint behavior. The regular API that CoppeliaSim provides functions to read the joint positions directly, simulating an absolute encoder response.

**Figure 3:**
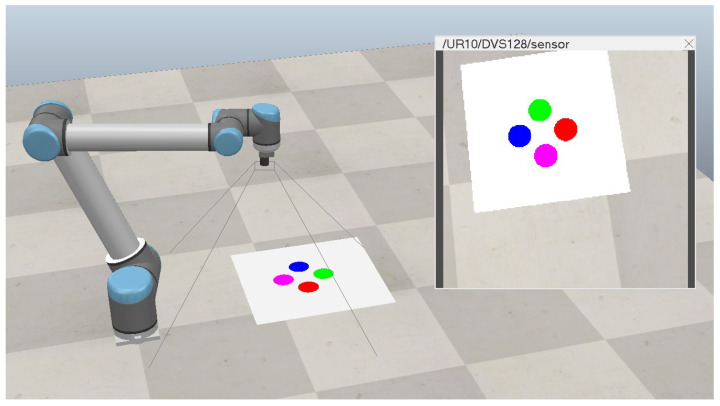
Scenario with UR10 simulation and colored circles in the camera vision.

Figure 3 depicts the main simulation scenario, where the robot performs the UVS task. Four distinctly colored circles are positioned on the ground, with the robot camera aiming at them. The XY image coordinates of each circle are detected using image processing, resulting in a total of 8 features. Noise affecting one of the features does not interfere with the measure of others.

The camera object used in simulations is a visual sensor that provides perspective imaging with a resolution of 256 × 256 pixels. That camera object is based on the DVS128 event camera preset model from the CoppeliaSim simulator but with a customized resolution, providing an equilibrium of quality and low latency imaging. It returns an OpenCV-compatible object through the API at each timestep. This allows the implementation of image processing filters directly in the Python controller application. All scenarios were configured with a timestep of 0.05 s, executed in the *stepping mode*.

### 5.2. Simulated Noise

For each feature, an independent additive noise following a Lévy α-stable distribution simulates unstructured environment problems that may happen to feature tracking. That distribution is a subset of the stable distribution and shares characteristics with the Gaussian distribution, which becomes a special case of it [26]. A convenient way to compute it was presented in [27], which introduced its characteristic equation as Equation (46).

(46) $$\log\phi (t)=\left\{\begin{array}{cc} -\gamma^{\alpha }{\left|t\right|}^{\alpha }\left\{1-i\beta \mathrm{sign}\left(t\right)\tan\frac{\pi \alpha }{2}\right\}+i\mu t, & \mathrm{if}\;\alpha \neq 1\\ -\gamma |t|\{1+i\beta \mathrm{sign}\left(t\right)\frac{2}{\pi }\log t\}+i\mu t, & \mathrm{if}\;\alpha =1 \end{array}\right..$$

Parameters μ∈R and γ>0 define, respectively, the displacement and the scale, which may be translated to mean and standard deviation in a Gaussian distribution case. The β∈[−1,1] parameter defines the skewness or symmetry of the distribution, so if β=0, the distribution graph is symmetrical. Parameter α∈(0,2] is the characteristic parameter, as it is related to the number of impulsive values in the distribution. If α=2, the Gaussian normal distribution appears, and as it lowers, the probability of appearing a number very distant to μ greatly increases.

**Figure 4:**
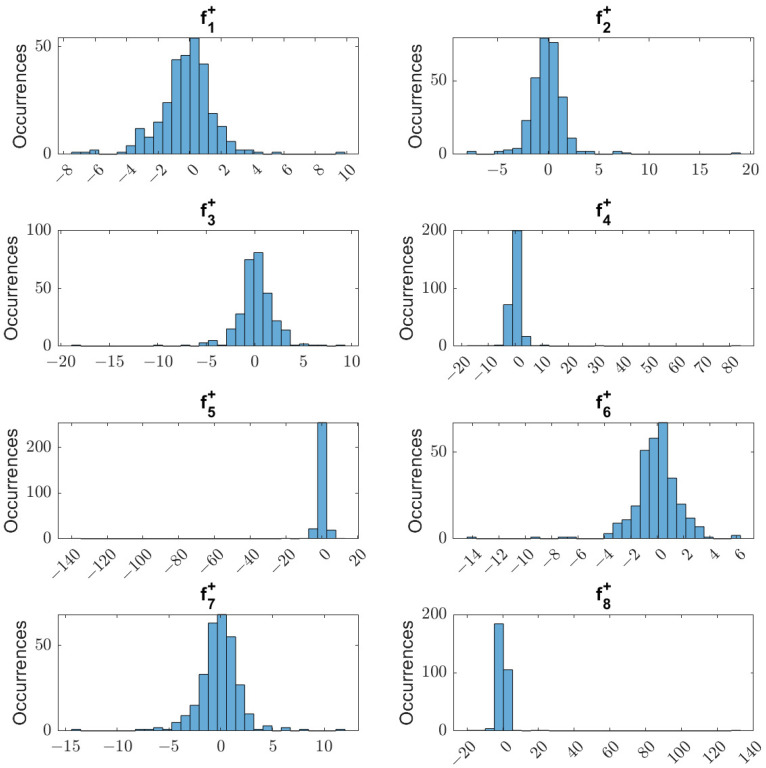
Additive noise histogram for each of the 8 features in an experiment using α=1.5455.

A normalized symmetric distribution is used for the experiments in this study, which means β=0, μ=0, and γ=1. When α=2, the additive noise is a white noise contaminating the feature tracking. Amn α<2 generates an additive noise that sometimes takes the value of the feature far off its detected place, in an impulse, and goes back to a value close to the rightly detected. Figure 4 shows the noise profile for an experiment with α=1.5455. Considering that we have 8 image features in our IBVS problem, $f_{i}^{+}$ are the random variables generated using alpha-stable distribution, producing additive noise to each feature. Their histograms differ because these variables are independent since different features should present different noise values at any simulation time. In the presented case, the impulsive noise is particularly intense in the fourth, fifth, and eighth features, causing a displacement of more than 80 pixels from the true value.

### 5.3. Monte Carlo Scenarios

Considering that α is the characteristic parameter that regulates the behavior of the additive noise on feature tracking, it is the main changing parameter in the simulation scenarios. A Monte Carlo series of experiments is developed with 12 linearly spaced α values varying between 1 and 2, as α=1 already presents a large number of outliers. For each α, $10^{2}$ independent UVS experiments are carried out. A total of four estimation techniques are implemented and compared in those experiments: traditional Kalman Filter (KF), Maximum Correntropy Kalman Filter (MCKF), Improved Maximum Correntropy Criterion Kalman Filter (IMCC-KF), and the proposed RMCKF for IBVS. All correntropy-induced techniques are using the simulated annealing approach.

This work studies three scenarios of performance comparison, which represent common IBVS feature regulation tasks. The first scenario compares all four techniques in a features translation IBVS task. The manipulator starts in the joint configuration $q=\{0,\;-\frac{\pi }{8},\;\frac{5\pi }{8},\;0,\;-\frac{\pi }{2},\;0\}$, placing the features in the top side of the camera image. Then, the visual servoing algorithm takes the task of centering the image features to the vector $f_{d}=\left\{149,\;145,\;125,\;121,\;101,\;145,\;125,\;169\right\}$. Although the $10^{2}$ experiments for each α have the same starting conditions, the noise distribution differs for each experiment, resulting in distinct results.

The second scenario differs from the first because each experiment starts with different joint configurations, maintaining all features in the camera view at the beginning of the simulation. Because of that, to reach the same image features desired vector, the IBVS is supposed to perform translation and rotation of the features. Lastly, the third scenario is the same setup as the second, but only the simulated annealing on the RMCKF is being evaluated compared to the fixed kernel bandwidth approach. The Kalman Filter is being used as a baseline of discussion.

The Integral of Time-weighted Absolute Error (ITAE) criterion is used as a performance metric. As 8 features are used, the scenario stores a vector norm of the ITAE results. After all $10^{2}$ experiments are run, the mean and standard deviation are persisted and shown in an error bar plot, where techniques are compared. Also, the initial value for the state vector x is computed using an analytical jacobian approximation using the starting robot joint positions and an initial guess for the image iteration matrix based on a priori camera calibration parameters. Afterward, the estimation algorithm evolves the state vector and the jacobian values.

## 6. Results and Discussions

This section presents the results of the three Monte Carlo experiment scenarios, and some discussion to explain the performance of the techniques. The first scenario compares the estimation algorithms in a simple feature translation task. The second scenario is harder than the first, because the robot starts at different configurations, requiring translation and rotation of the features. Whereas in previous scenarios, all techniques use simulated annealing, the third scenario evaluates the relevance of the simulated annealing approach for the kernel bandwidth, compared to tests with a fixed kernel bandwidth.

### 6.1. Scenario 1

**Figure 5:**
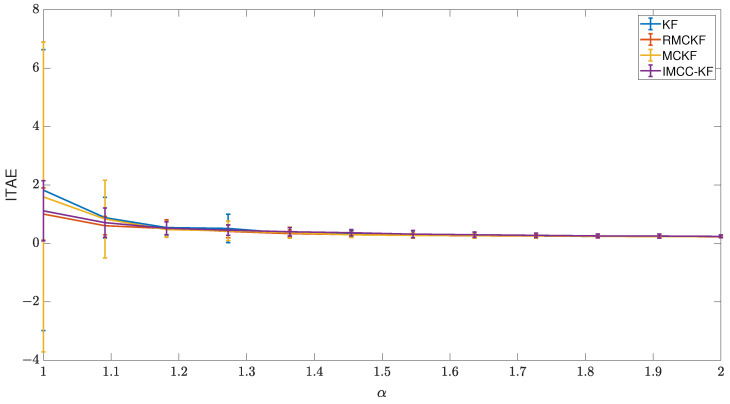
Comparison of techniques performances on translation task.

Figure 5 shows the results of the first scenario. When the noise distribution profile is Gaussian, i.e., α=2, there is no relevant difference between the techniques, which was expected since the Kalman Filter is optimal for problems involving white noise signal. However, more impulsive noise appears for α closer to one, and the correntropy-induced techniques perform better in those situations. In the designed scenario, the advantages of RMCKF over the remaining techniques are shown by the lines representing the mean of ITAE and the standard deviation bar, representing UVS tasks being resolved with less accumulated error and more homogeneity.

### 6.2. Scenario 2

**Figure 6:**
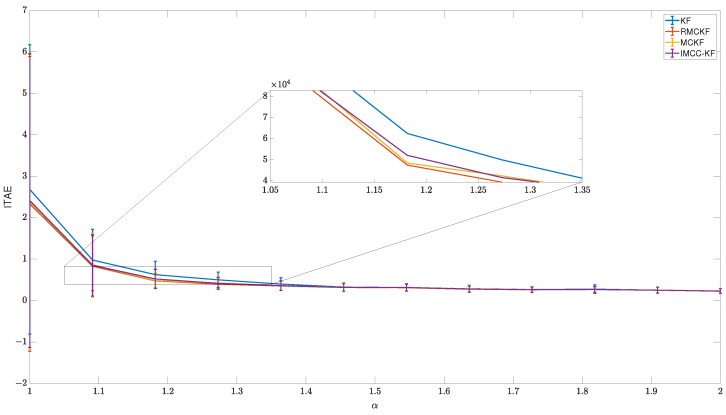
Comparison of techniques performances under changing starting configurations.

The results for the second scenario are shown in Figure 6. The tasks required by that scenario are more difficult for all techniques, as the mean ITAE lines are generally higher than those in the previous scenario results, especially for the highly impulsive noise experiments. The zoom plot aids in showing that the RMCKF performs better than the other approaches. As the robot starting configuration changes over experiments, the standard deviation bar does not present the same effect from the previous scenario. Still, it shows that the algorithm performances are not too far from the mean, except for the α=1 experiments, where some cases fail to center the image features due to a combination of joint configuration and noise leading to out-of-image features.

The better performance of RMCKF is because not all image feature measures present impulsive values simultaneously, so it tries to estimate the jacobian and perform the visual servoing task concisely with the remaining values. The other correntropy-induced techniques skip a new jacobian estimation but still use the old estimation for an IBVS control action, which may lead to incorrect joint motion since the value of both image and kinematics jacobian are highly dependent on the system’s current configuration. The Kalman filter algorithm tries to correct the estimation at all times, even when the image processing algorithm states that the feature is very far from its previous position, e.g., out of the image. However, when only Gaussian noise is expected, KF is the preferred option, because of its simpler implementation.

### 6.3. Scenario 3

**Figure 7:**
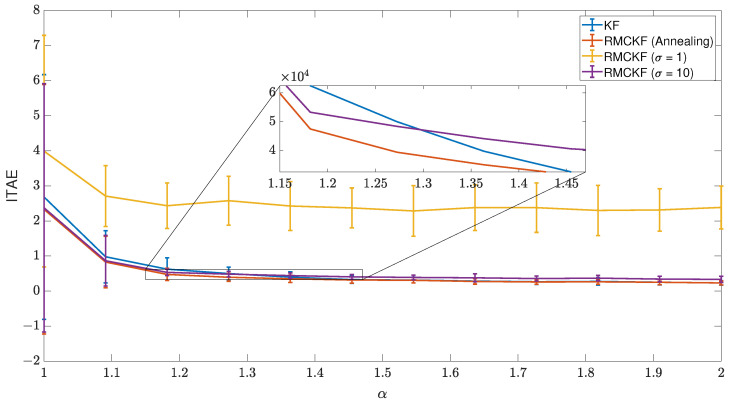
Comparison of RMCKF using different kernel bandwidth approaches.

Figure 7 depicts the results of the third scenario. The core concept to understand the importance of the simulated annealing approach here is understanding how the estimation error e(k) evolves from the start to the end of the task. It is important to remember that e(k) is the difference between the *R*-normalized measured feature motion Z(k) and the estimated feature motion H(k)x(k|k−1) based on joint movement. Thus, even with an initial jacobian guess, e(k) may be big enough at the start of the IBVS task, requiring higher estimation correction. If the kernel bandwidth σ is too small, the algorithm may consider that estimation error originated by a measure outlier, skipping the contribution of the components related to a specific feature.

The RMCKF with fixed σ=1 is very intolerant to big estimation changes, leading to poor performance in the IBVS task, worse than KF. On the other hand, the RMCKF with fixed σ=10 performs better than KF for very impulsive noise, with results close to the simulated annealing RMCKF. However, as the noise distribution starts to present a more Gaussian aspect, the advantages of the RMCKF with fixed σ=10 to the KF disappear, so at α close to 2, the performance of KF is better. More precisely, their performance lines cross at α around 1.3, as shown by the zoom plot in Figure 7.

The simulated annealing RMCKF has the best performance of the compared approaches. In the execution of tasks, it is tolerant initially, as the algorithm expects bigger changes in the estimation correction and retains the impulsive noise rejection for the rest of the assignment. If a measure outlier appears at the end moments of the simulation, the technique still rejects it, but the convergence from the estimation for the remaining features motion should be sufficiently reliable to still perform the task. When the noise profile is closer to a Gaussian distribution, it works similarly to the Kalman filter.

## 7. Conclusions

This work presented a new approach to handling non-Gaussian noise in uncalibrated image-based visual servoing tasks. That type of noise is common when feature tracking in unstructured environments, where light, reflexes, and occlusions are not in control. The proposed RMCKF was designed specifically for the IBVS problem, managing the ways on which both the estimation and the control action are calculated, so the robot may perform its tasks with as much information as it can rely upon, even when some of the measures are potentially impulsive outliers. Extensive experiments were driven using random configuration and noise parameters. The results showed that the proposed approach, using the simulated annealing, outperformed the compared techniques in the tested scenarios.

The studied and proposed approaches were correntropy-induced techniques, which took advantage of the characteristics of the kernel-based correntropy criterion in their equations to reach a desired behavior when outliers are present. However, an extended study on how the Maximum Correntropy Criterion can deeply incorporate estimation algorithms is required to explore other characteristics of the involved higher-order statistical moments that are evolving with the estimation.

Impulsive noise in the UVS scenario can possibly appear, when there is fast external interference in the observed objects. However, another case is when something occludes the object momentarily, retaining that noise value for a longer time, and presenting its own dynamic. The correntropy-induced techniques can be robust to impulsive noise, but in the latter case, they are not known to contribute to the task.

Future work will apply the proposed technique to different robot models and mechanical structures to explore the limits of the IBVS jacobian estimation. Also, the RMCKF can be adapted to directly estimate the pseudoinverse IBVS jacobian, skipping a matrix inversion in the control action, but the convergence, advantages, and side effects of that approach are yet to be studied.

## Figures and Tables

**Table 1:** Denavit–Hartenberg parameters for the UR10 manipulator.

i	$\theta_{i}$	$d_{i}$	$a_{i}$	$\alpha_{i}$
1	$\theta_{1}$	0.128	0	−π/2
2	$\theta_{2}-\pi /2$	0	0.6127	0
3	$\theta_{3}$	0	0.5716	0
4	$\theta_{4}-\pi /2$	0.1639	0	−π/2
5	$\theta_{5}$	0.1157	0	π/2
6	$\theta_{6}+\pi /2$	0.0922	0	0

## Data Availability

All results in csv data are available at [28]. The source code with simulation scenarios is available only at the repository https://github.com/AI-SPARC/uncalibrated-visual-servoing (accessed on 7 September 2023).

## Abbreviations

The following abbreviations are used in this manuscript:

IBVS	Image-Based Visual Servoing
IMCC-KF	Improved Maximum Correntropy Criterion Kalman Filter
ITAE	Integral of Time-weighted Absolute Error
KF	Kalman Filter
MCC	Maximum Correntropy Criterion
MCKF	Maximum Correntropy Kalman Filter
RMCKF	Regularized Maximum Correntropy Kalman Filter
UVS	Uncalibrated Visual Servoing
